# Functional Brain Controllability Alterations in Stroke

**DOI:** 10.3389/fbioe.2022.925970

**Published:** 2022-06-27

**Authors:** Xuhong Li, Feng Fang, Rihui Li, Yingchun Zhang

**Affiliations:** ^1^ Department of Rehabilitation Medicine, The Third Xiangya Hospital, Central South University, Changsha, China; ^2^ Department of Biomedical Engineering, University of Houston, Houston, TX, United States; ^3^ Center for Interdisciplinary Brain Sciences Research, Department of Psychiatry and Behavioral Sciences, Stanford University School of Medicine, Stanford, CA, United States

**Keywords:** stroke, brain controllability, motor control, EEG, fNIRS (functional near infrared spectroscopy)

## Abstract

Motor control deficits are very common in stroke survivors and often lead to disability. Current clinical measures for profiling motor control impairments are largely subjective and lack precise interpretation in a “control” perspective. This study aims to provide an accurate interpretation and assessment of the underlying “motor control” deficits caused by stroke, using a recently developed novel technique, i.e., the functional brain controllability analysis. The electroencephalography (EEG) and functional near-infrared spectroscopy (fNIRS) were simultaneously recorded from 16 stroke patients and 11 healthy subjects during a hand-clenching task. A high spatiotemporal resolution fNIRS-informed EEG source imaging approach was then employed to estimate the cortical activity and construct the functional brain network. Subsequently, network control theory was applied to evaluate the modal controllability of some key motor regions, including primary motor cortex (M1), premotor cortex (PMC), and supplementary motor cortex (SMA), and also the executive control network (ECN). Results indicated that the modal controllability of ECN in stroke patients was significantly lower than healthy subjects (*p* = 0.03). Besides, the modal controllability of SMA in stroke patients was also significant smaller than healthy subjects (*p* = 0.02). Finally, the baseline modal controllability of M1 was found to be significantly correlated with the baseline FM-UL clinical scores (*r* = 0.58, *p* = 0.01). In conclusion, our results provide a new perspective to better understand the motor control deficits caused by stroke. We expect such an analytical methodology can be extended to investigate the other neurological or psychiatric diseases caused by cognitive control or motor control impairment.

## Introduction

Stroke is the major cause of motor impairment, leading to motor control deficits at acute stage (Langhorne et al., 2011). More than 1.1 million people in the United States report difficulty with functional limitations in daily lives following stroke (Inman et al., 2012). Accurate interpretation and identification of motor impairment after stroke are of cardinal importance for the patient, clinician, and healthcare system (Bonkhoff et al., 2020). Over the past decades, effort has been taken to understand the underlying neural control mechanisms related to motor impairment following stroke to enhance the treatment efficacy of stroke rehabilitation interventions (Collin and Wade, 1990; Mani et al., 2013; Vliet et al., 2020). Emerging evidences have shown that various brain regions are specialized for different aspects of motor control (Mani et al., 2013), indicating it is critical to precisely define and evaluate the controllability of different brain regions that contribute to specific motor control deficits caused by stroke. Unfortunately, such a precise evaluation of motor control deficits of stroke, in which both high resolution brain imaging strategy and accurate description of “controllability” are needed, is not currently available.

Recently, advanced neuroimaging techniques, including functional magnetic resonance imaging (fMRI), functional near-infrared spectroscopy (fNIRS), and electroencephalography (EEG), have been widely employed to investigate the dynamic alteration of cortical excitability and network connectivity following stroke, and shown great potential to understand the relationship between the dysfunctional brain network and motor control deficits (Grefkes et al., 2008a; Bajaj et al., 2014; Snyder et al., 2021). For example, previous fMRI study illustrated that the motor control deficits of stroke patients were associated with pathological intra- and inter-hemispheric interactions among key motor regions such as primary motor cortex (M1), premotor cortex (PMC), and supplementary motor cortex (SMA), and executive control network (ECN) (Grefkes et al., 2008a; Zhao et al., 2018). A recent study employing EEG to investigate the resting-state networks under different frequency bands in stroke showed that reduced cortical activity and connectivity in alpha and beta bands in stroke patients might explain the motor impairment caused by stroke (Snyder et al., 2021). Similarly, a previous fNIRS study applying the spectral interdependency methods demonstrated the bi- and uni-directional connectivity between motor brain regions were associated with specific movement suppression and motor control execution, and could provide promising biomarkers to characterize motor control impairment in stroke patients (Bajaj et al., 2014).

While unimodal fMRI, fNIRS, and EEG studies have provided critical insight into the brain network alteration associated with stroke, their limitations have prevented in-depth study to simultaneously extract the spatial and temporal information of the brain activity in a good precision. Specifically, EEG offers high temporal accuracy to unveil the dynamics of neural activity but suffers from the volume conduction problem, which may make the estimation of brain connectivity unreliable (Winter et al., 2007). FMRI and fNIRS show higher spatial resolution to locate the brain activity than EEG (spatial resolution: fMRI > fNIRS > EEG), however, these two neuroimaging techniques are incapable of recovering accurate time course of cortical activity and the accuracy of hemodynamic-based connectivity network is questionable (Roebroeck et al., 2011). To overcome these limitations, a recently developed, spatiotemporal specific method, dynamic brain transition network (DBTN), for EEG and fNIRS (or fMRI) integration analysis was applied to reconstruct highly specific patterns of cortical activity, which were then used to recover the general and conditionally-specific brain networks that support stimulus response (Nguyen et al., 2019; Fang et al., 2020). Previous study has utilized the DBTN source imaging approach to identify biomarkers associated with motor function recovery and document the post-stroke motor reorganization (Li et al., 2020). The results showed that the functional brain connectivity of PMC, M1, and SMA were potential biomarkers to assess the motor function recovery of stroke, and the DBTN source imaging strategy was potentially useful for monitoring and predicting post-stroke motor recovery (Li et al., 2020).

**TABLE 1:** Participants demographics and clinical characteristics.

Patients ID	Age (years)	Sex (F/M)	Affective side	Days after stroke	Lesion location	FM-UL	
						Pre	Post
01	55	Male	R	45	Left basal ganglia	12	\
02	66	Female	R	89	Left pons	18	33
03	36	Male	R	75	Left basal ganglia	30	\
04	46	Male	R	40	Left thalamus	53	\
05	37	Male	R	84	Left coronal radiate	32	\
06	55	Female	R	32	Left pons	56	\
07	61	Female	R	42	Left basal ganglia	14	\
08	47	Male	R	72	Left basal ganglia	20	\
09	36	Male	R	99	Left basal ganglia	17	\
10	43	Male	R	101	Left basal ganglia	18	20
11	63	Female	R	52	Left pons	16	\
12	40	Male	R	56	Left basal ganglia	61	\
13	56	Male	L	62	Right basal ganglia	56	60
14	51	Female	L	44	Right basal ganglia	43	49
15	50	Male	L	32	Right basal ganglia	11	13
16	43	Male	L	110	Right basal ganglia	22	27

Even though previous studies have reported potential biomarkers to assess the motor control deficits of stroke, all these biomarkers themselves are not directly associated with the “control” assessment of the brain. As such, a specific understanding of the “motor control” deficits caused by stroke, which may lead to advanced interpretation of the physiological symptom observed in stroke patients, is remains lacking. Recently, network control theory has been applied to interpret brain state transitions (Gu et al., 2015). Conventional graph-based measures show the local properties of varied brain regions and their important roles in their network architectures (Sporns, 2018). Differently, control theory-based network measures describe one brain region’s capability to change the brain behavior from one state to another state (Gu et al., 2015). For example, modal controllability diagnostic describes the ability of one brain region to steer the brain networked system into difficult-to-reach state (Gu et al., 2015). Previous study has employed the brain controllability analysis to assess the cognitive control deficit in neurological and psychiatric diseases such as depression and dementia (Fang et al., 2021). However, no study has ever utilized brain controllability measure to assess the motor control deficit of stroke, to specifically describe the “motor control” deficit with a specific “controllability” measurement.

In this study, we integrated our recently developed DBTN-based fNIRS-informed EEG source imaging approach, and functional brain controllability analysis to assess “motor control” deficits caused by stroke. We hypothesized that the modal controllability of the key motor brain regions (M1, PMC, and SMA) and the ECN would decrease among stroke patients compared to healthy subjects. To the best of our knowledge, this study represents the first effort to employ the brain network “controllability” diagnostic to specifically interpret the “motor control” deficits caused by stroke. Additionally, this study is also the first study to apply the brain controllability analysis based on the non-invasive, portal, and costless neuroimaging tools with a high spatiotemporal fNIRS-informed EEG source imaging approach.

## Materials and Methods

### Study Design

Sixteen stroke patients with hemiparesis (5 females and 11 males; age 49.1 ± 9.4 years) were recruited from Guangdong Provincial Work Injury Rehabilitation Center, and 11 age-matched, healthy subjects (3 females and 8 males; age 41.2 ± 15.8 years) were recruited as the control group. All participants are right-handed. The experimental protocol was approved by the ethics committee of the Guangdong Provincial Work Injury Rehabilitation Center (AF/SC-07/2016.30). Participants gave written informed consent according to the Declaration of Helsinki.

The inclusion criteria for stroke patients were as follows: 1) stroke that occurred 1–6 months prior to the first assessment, 2) age between 18 and 70 years, and 3) able to follow instructions and to consent (Mini Mental State Examination score >27). The exclusion criteria were as follows: 1) deficits in communication or attention that would interfere with the experiment participation, 2) contraindication to MRI scanning, and 3) other diseases that would substantially affect the function of upper extremity.

All patients underwent a 4-weeks conventional rehabilitation intervention in the hospital. The intervention included standard physical training (walking, sitting, standing balance, and movement switching), occupational therapy (eating, drinking, swallowing, dressing, bathing, cooking, reading and writing, and using the restroom), and massage for 6 h per day, 5 days per week. Prior to the beginning of intervention, all patients underwent a baseline assessment of upper extremity function by Fugl-Meyer Assessment rating scale (FM-UL, normal = 66) and participated in a concurrent EEG-fNIRS recording (pre-intervention) (Gladstone et al., 2002). Ten patients were not able to complete the entire rehabilitation intervention and thus, were ineligible to participate in the post-intervention EEG-fNIRS recording and clinical assessment. Therefore, only six patients participated in the concurrent EEG-fNIRS recording and clinical assessment of motor function in the post-intervention session. All motor function assessments were performed by an experienced therapist from the Department of Rehabilitation Medicine in the hospital.

### Experimental Paradigm

**FIGURE 1:**
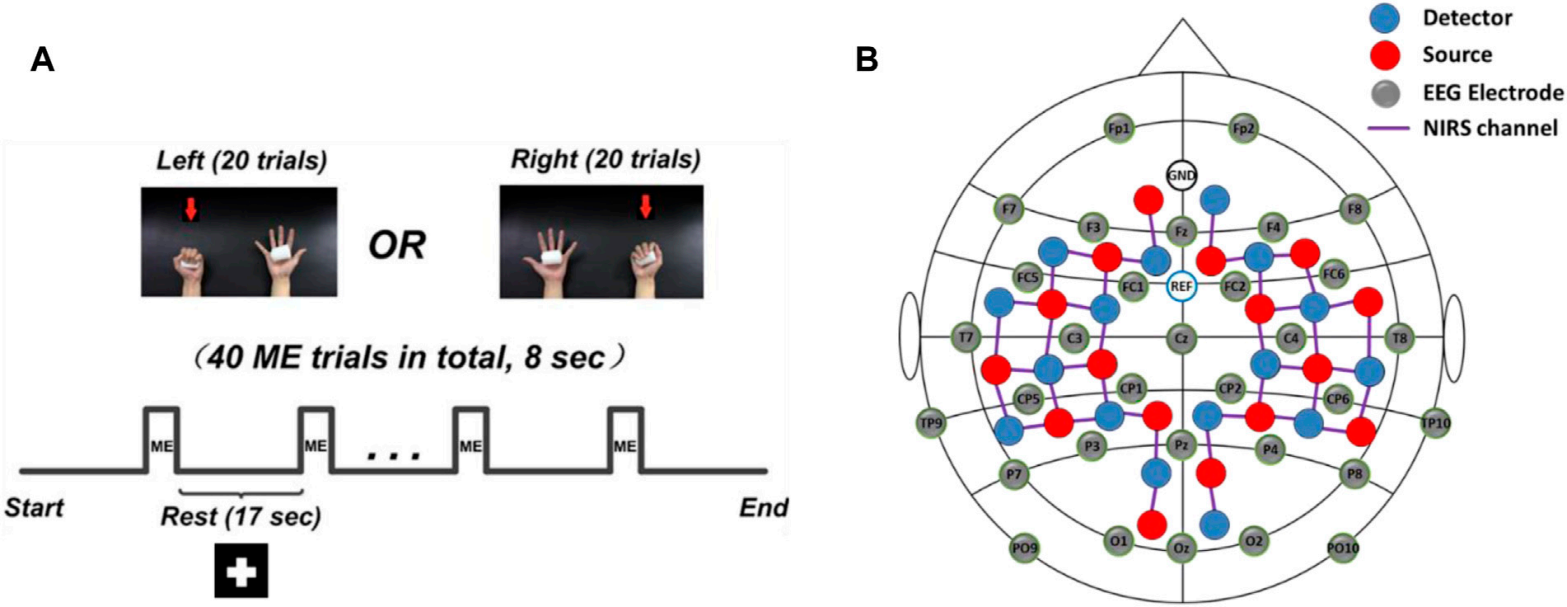
Experimental design. **(A)** The experimental motor executive task used in the study. The “ME” represents motor execution. **(B)** The EEG and fNIRS channel locations.

During the experiment, participants received visual instruction through a monitor placed in front of them. A motor executive (ME) paradigm consisted of 40 randomized trials of left- and right-hand clench tasks (20 trials for each hand) was employed. Each trial started with an 8-s ME task, indicated by a “+” symbol in a black background (Figure 1A). During the ME period, subjects were asked to naturally squeeze a sponge ball with the corresponding hand shown on the monitor. Patients were required to try their best to squeeze the sponge ball using their affected hands without causing any shaking of their bodies. In this study, the whole-hand clenching task was applied since previous studies reported that the whole-hand clenching evoked stronger brain cortical activations than classic motor task such as finger tapping (Grefkes et al., 2008b). Meanwhile, the whole-hand clenching task is relatively easier to be executed by stroke patients who have motor deficits.

### Data Acquisition

A concurrent EEG and fNIRS recording paradigm was employed to collect the EEG signal and hemodynamic response signal (Figure 1B). Specifically, 32 active EEG electrodes were placed on the scalp, and the EEG signals were measured using an EEG recording system (Brain Products GmbH, Germany) with 500 Hz sampling rate. Meanwhile, a total of 40 fNIRS channels were positioned over the main brain regions, including the motor cortex, frontal cortex, temporal cortex, and occipital cortex. FNIRS signals were recorded simultaneously using a continuous-wave NIRS imaging system (NIRScout, NIRx Medizintechnik GmbH) with 3.91 Hz sampling rate.

### EEG fNIRS Preprocessing

**FIGURE 2:**
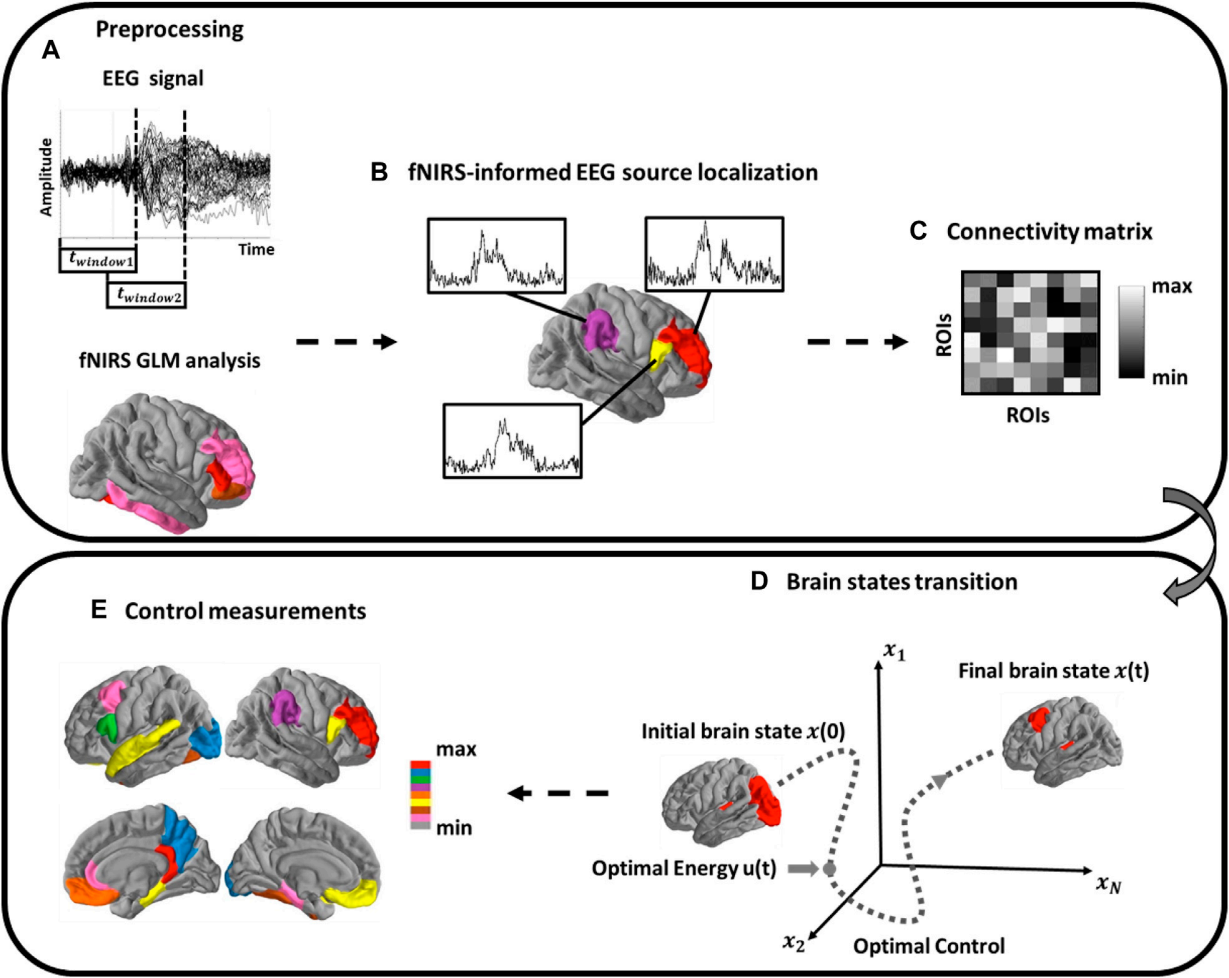
Schematic of Methods. **(A)** EEG analysis using a sliding window scheme. FNIRS activation map is then extracted to form spatial priors; **(B)** Source localization analysis of cortical source activity; **(C)** Functional brain network construction; **(D)** Estimation of brain network dynamic process and the brain controllability analysis; **(E)** Calculate the modal controllability of each single brain region.

The analytical pipeline is shown in Figure 2. The raw EEG signals were first filtered by a notch filter at 50 Hz to remove powerline noise and then a fourth-order Butterworth bandpass filter (0.5–45 Hz). Eye movement artifact was then removed using independent component analysis (ICA) strategy. The common average method was utilized to re-reference the EEG signals (Ludwig et al., 2009). After that, EEG signals were segmented into multiple trails that began 2000 ms before the task onset and ended 8000 ms after the task onset, and baseline correction was performed for each trial. Finally, we manually inspected and excluded any trial with large artifact.

For the fNIRS signals, a fourth-order Butterworth band pass filter (0.01–0.5 Hz) was applied first to eliminate artifacts such as cardiac interference (0.8 Hz). Following this, motion artifacts were removed from the fNIRS signals using a wavelet-based method (Molavi and Dumont, 2012). The concentration changes of the HbO and HbR were then computed utilizing the modified Beer-Lambert Law (Ferrari and Quaresima, 2012). The obtained signals were manually inspected for every channel, wherein trials with apparent spikes and discontinuous segments were deemed as noisy trials and excluded from further analysis (usually signal changes with amplitude >0.4 au and exceeding a threshold of 100 in change of standard deviation within 0.3 s) (Delgado Reyes et al., 2018). Finally, the general linear model (GLM) was employed to obtain the activated channels that significantly induced by each hand movement, which would be used as spatial priors for the EEG source imaging.

### fNIRS-Informed EEG Source Localization

#### Forward Calculation

In this study, the MNI 305 template was used as common brain model for all subjects (Fonov et al., 2011). The high-density cortical layer and the brain-skull-scalp layers were generated on the brain model using the Freesurfer analysis suite (Fischl, 2012). The boundary element method (BEM) was then employed to construct the 3-layer brain model (Fuchs et al., 2002). A lead-field matrix G was then computed based on the cortical source space, the 3-layer brain model, and the 32 EEG channels *via* forward calculation (Hallez et al., 2007).

#### Inverse Calculation

Our recently developed high spatiotemporal fNIRS-constrained EEG source imaging approach, DBTN, was employed to perform source analysis (Nguyen et al., 2016; Nguyen et al., 2018; Li et al., 2020). Following this method, electrical activity within the source space is reconstructed based on multimodal, sliding-window calculations, which makes the algorithm spatially precise and resilient to depth bias and noise from volume conduction (Nguyen et al., 2016; Nguyen et al., 2018; Li et al., 2020). Briefly, the calculation of the current density J can be formulated as:

$$J=RG^{T}{\left(GRG^{T}+\;\lambda^{C}\right)}^{-1}Y$$ (1)

where Y represents the EEG signals and J indicates the unknown source activity. C and R represent the noise and source covariance matrices, respectively. The regularization parameter λ represents a trade-off between the model accuracy and complexity that is traditionally determined through the L - curve method. Within this construction, the source covariance matrix, R , represents prior knowledge about the distribution of J . Under the framework of the high spatiotemporal fNIRS-constrained EEG source imaging (DBTN), however, R is constructed as a weighted sum of the active spatial priors, where each individual prior is a sub-map of the fNIRS activation pattern, as mentioned above:

$$R=\sum_{i=1}^{N}\lambda_{i}^{R}Q_{i}$$ (2)

Following this equation, R is defined by the sum of N covariance components Q = ( $Q_{1},\;\ldots ,\;Q_{N}$ ), weighted by an unknown hyperparameter $\lambda^{R}$ . Each individual covariance component, $Q_{i}$ , is formed from a subset of the fNIRS map. The hyperparameters $\lambda^{R}$ were estimated for each EEG window using a Restricted Maximum Likelihood algorithm (Nguyen et al., 2016), and the corresponding current densities were calculated. The DKT40 atlas was then employed to form 62 regions of interest (ROIs) (Klein and Tourville, 2012). More details about the DBTN methodology can refer to (Nguyen et al., 2016; Nguyen et al., 2018; Li et al., 2020).

### Functional Brain Network Controllability Analysis

#### Functional Network Construction

DBTN-based source localization formed a basis multivariate time-series for subsequent functional connectivity analysis using a measure of weighted phase lag index (wPLI) (Vinck et al., 2011). The wPLI method is a data-driven technique based on the weighted phase differences between two time-series signals. The functional brain network was then constructed by the wPLI values and utilized for the following brain network controllability analysis.

#### Brain Controllability Analysis

One of the critical steps in applying network control theory to the human brain is to define a model for the dynamics of neural processes (Gu et al., 2015; Karrer et al., 2020). In this study, a simplified, noise-free, linear, and time-invariant model was employed to build the brain network dynamic model (Gu et al., 2015). The model equation can be formulated as follows:

x(t+1)=Ax(t)+Bu(t) (3)

where x describes the state (that is, the magnitude of neurophysiological activity) of brain regions over time, and A is the functional connectivity matrix constructed by the wPLI method. The input matrix B specifies the control nodes and the input u denotes the external stimulation. In this study, the external stimulation of u can be considered as the experimental paradigm shown on the screen that elicited the preceding of motor behaviors in the brain.

The modal controllability was then utilized to evaluate the control capability of various regions in steering the network system into different ease level of states (Medaglia et al., 2017).The modal controllability reflects the ease of a node to push the brain network system into many different difficult-to-reach states (Medaglia et al., 2017). Mathematically, it was defined as:

$$\phi_{i}=\;\sum_{j=1}^{N}\left(1-\;\lambda_{j}^{2}\left(A\right)\right)v_{ij}^{2}$$ (4)

$v_{ij}$ is the element of the eigenvectors matrix of A and $\lambda_{j}$ is the jth vij is the element of the eigenvectors matrix of A and λj is the jth eigenvalue.

From a cognitive perspective, the brain areas with high modal controllability may be important in switching the brain between many cognitive functions that require significant cognitive effort (Gu et al., 2015). If control energy can be likened to cognitive effort and if brain states can be likened to cognitive functions, then the difficult-to-reach state refers to the brain state that requires significant cognitive effort to reach from the initial brain cognitive state such as from a resting brain state to a motor performance state that is cognitively demanding. In this study, we calculated the modal controllability of the three main motor brain regions, M1, PMC, and SMA, from the contralateral sides, and also the psychological brain system of ECN for both stroke patients and healthy subjects. In this study, the ECN was extracted from the ROIs located in the prefrontal cortex including the ventromedial prefrontal cortex and dorsolateral prefrontal cortex, parietal cortex, and anterior cingulate cortex (Callejas et al., 2005; Duncan, 2013; Dong et al., 2015).

### Statistical Analysis

Linear regression analysis was first performed to investigate the relationship between the modal controllability and the node strength (Montgomery et al., 2021). The modal controllability of three different motor brain regions, M1, PMC, and SMA, were computed and compared, respectively, between stroke patients and healthy controls using non-parametric statistical test, Mann Whitney U test (Nachar, 2008). The modal controllability of ECN was also compared between stroke patients and healthy controls using Mann Whitney U test. The baseline modal controllability of M1, PMC, SMA, and ECN were correlated with the baseline clinical scores, FM-UL, using linear regression model. Meanwhile, the changes of modal controllability of the three motor-related regions and the ECN were also correlated with the changes of baseline clinical scores from pre- and post-intervention using linear regression model. False discovery rate (FDR) method was employed for correction of multiple comparisons (Genovese et al., 2002).

## Results

### Demographic and Clinical Behavior Data

Table 1 summarizes the demographic information of the stroke patients including age, gender, site of the lesion, time of stroke, and clinical assessment scores. Statistical analysis showed that there were no significant differences between stroke patients and healthy subjects in terms of age (*p* > 0.05, *t* test) and gender (*p* > 0.05, chi-square test) (Satorra and Bentler, 2001; De Winter, 2013).

### Controllability of Psychological Brain Network and Motor Brain Regions

**FIGURE 3:**
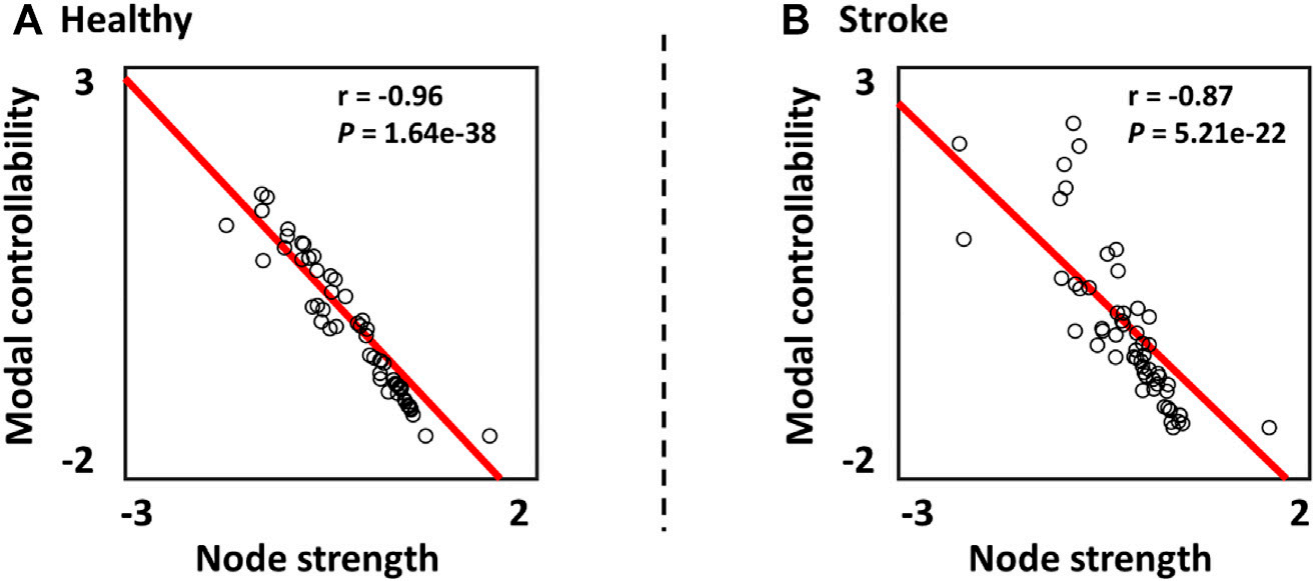
Relationship between the z-scored modal controllability and the z-scored node strength in **(A)** Healthy subjects and **(B)** Stroke patients.

In Figure 3, the relationship between the modal controllability and the node strength was investigated. The results showed that the z-scored modal controllability was significantly correlated with the z-scored node strength in both healthy subjects (*r* = −0.96, *p* = 1.62e-38) and stroke patients (*r* = −0.87, *p* = 5.21e-22). The negative correlation between the modal controllability and the node strength are consistent with previous studies (Gu et al., 2015; Wiles et al., 2017).

**FIGURE 4:**
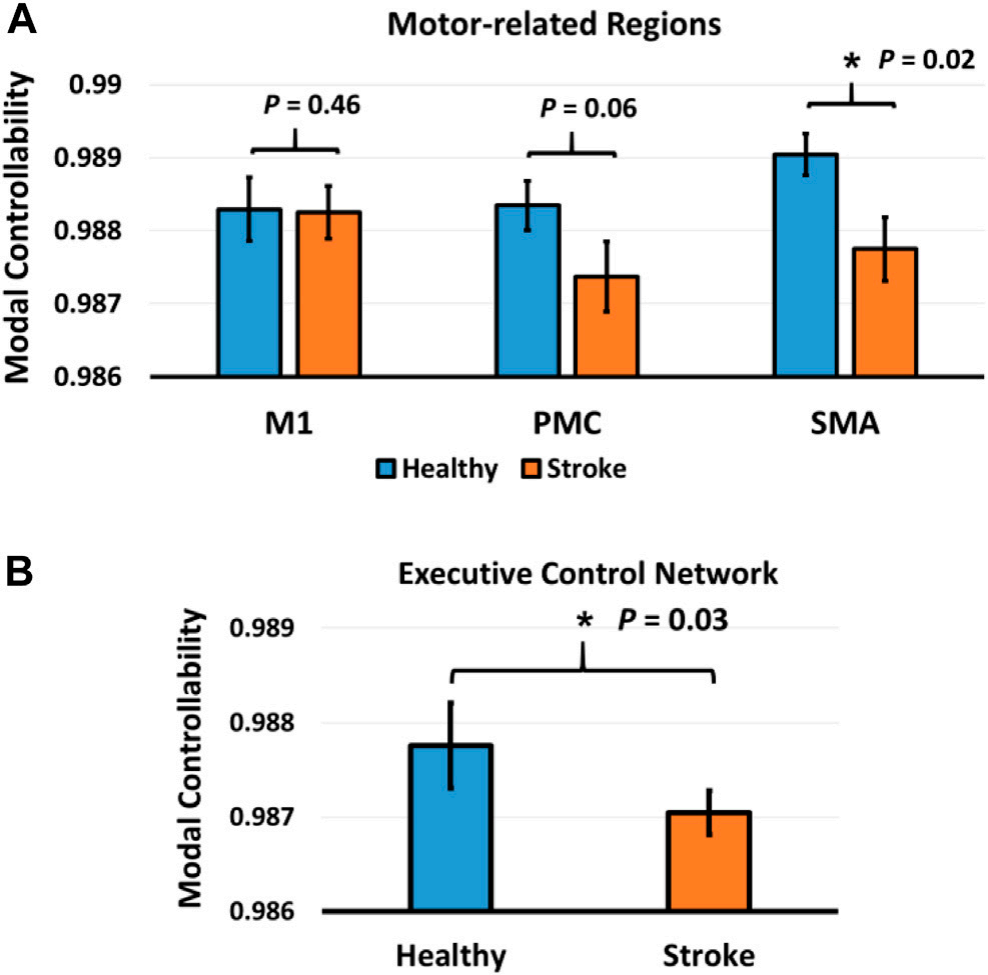
**(A)** Comparison of modal controllability in M1, PMC, and SMA between stroke patients and healthy subjects. **(B)** Comparison of modal controllability in executive control network (ECN) between stroke patients and healthy subjects. Asterisk represents significant difference (*p* < 0.05) after multiple comparison correction.

Then, the modal controllability of ECN was computed and statistically compared between the two groups. As shown in Figure 4B, the modal controllability of ECN in healthy subjects was significantly larger than the modal controllability of ECN in stroke patients (*p* = 0.03). Following this, the modal controllability of three key motor regions, M1, PMC, and SMA, were calculated and statistically compared. In Figure 4A, the modal controllability of SMA in healthy subjects was significantly higher than the modal controllability of SMA in stroke patients (*p* = 0.02, FDR-corrected). The modal controllability of PMC was significantly larger in healthy subjects than stroke patients before multiple correction (*p* < 0.05, uncorrected), but insignificant after multiple correction (*p* = 0.06, FDR-corrected). No significant difference of modal controllability in M1 was observed between stroke patients and healthy subjects (*p* = 0.46, FDR-corrected).

### Correlation Between Baseline Controllability and Clinical Scores

**FIGURE 5:**
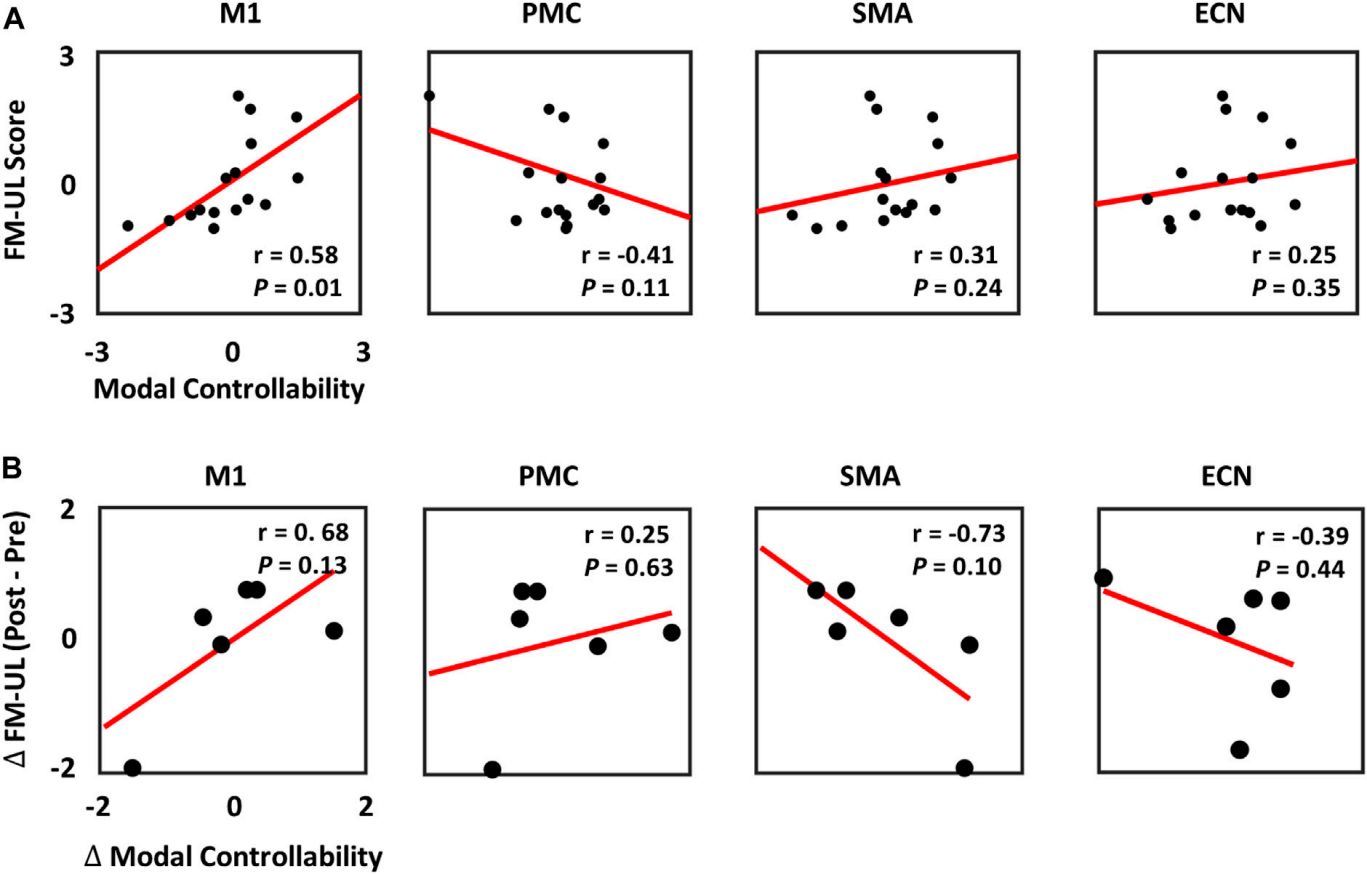
**(A)** Relationship between the baseline FM-UL scores and the baseline modal controllability in M1, PMC, SMA, and ECN. **(B)** Relationship between the changes of FM-UL scores and the changes of modal controllability at pre- and post-intervention among the 6 stroke patients.

The relationship between the baseline modal controllability and the baseline clinical scores (FM-UL) was then explored in stroke patients. The z-scored baseline modal controllability and FM-UL scores were computed and correlated using linear regression model. As shown in Figure 5A, the baseline modal controllability of M1 was significantly correlated with the baseline FM-UL scores (*r* = 0.58, *p* = 0.01). No significant correlation was observed between the FM-UL scores and the modal controllability of PMC, SMA, and ECN.

In order to identify biomarkers to predict the recovery rate of stroke patients, the changes of modal controllability and the changes of FM-UL scores at pre- and post-intervention recordings were calculated and correlated by the linear regression modal. In Figure 5B, the results showed that no significant correlation was observed between the changes of modal controllability and the changes of FM-UL scores based on six stroke patients’ data, even though very high correlation existed.

## Discussion

While current neuroimaging studies have proposed potential network-level biomarkers to assess the motor control impairment and better understand the underlying neural mechanisms on stroke patients (Grefkes et al., 2008a; Bajaj et al., 2014; Snyder et al., 2021), none of the biomarkers could provide a “control” concept to specifically describe the “motor control” deficits. Therefore, the primary goal of this study is to assess the “motor control” deficits of stroke patients by performing a high spatiotemporal resolution source imaging analysis, and employing a specific “control” diagnostic, which is the modal controllability (Gu et al., 2015). The main findings in this study are that the modal controllability of SMA and ECN are significantly lower in stroke patients than healthy subjects. In addition, the baseline modal controllability of M1 is found to be significantly correlated with the baseline clinical scores of stroke patients. To the best of our knowledge, this study represents the first attempt to apply the measure of “controllability” to specifically assess the “motor control” deficits caused by stroke. Besides, this is also the first study to employ the brain network controllability analysis based on the non-invasive, portable, and costless neuroimaging modalities associated with a high spatiotemporal fNIRS-informed EEG source localization approach (Nguyen et al., 2018). The methodologies utilized in this study may provide a new perspective to better understand the cognitive control or motor control impairment of different neurological or psychiatric diseases, and promote the development of neuromodulation strategies in an experimentally friendly manner.

In general, most stroke patients suffer from various degrees of motor deficits, which has been associated with the functional impairment across different motor control areas such as M1, PMC, and SMA (Zhao et al., 2018). The PMC and SMA brain regions are appear to be higher level areas that encode complex patterns of motor output and select appropriate motor plans to achieve desired end results, while M1 appears to be relatively lower hierarchy and decomposes movement into simple components in a body map, and these simple movement components are then communicated to the spinal cord for execution (Graziano, 2006). Previous study has employed brain connectivity analysis to assess the relationship between cortical disconnection and motor performance, demonstrating that the cortical disconnection of M1 and SMA are associated with the upper/lower extremity motor control performance of stroke patients (Peters et al., 2018). The results further show that the SMA is important in the temporal organization of movement and becomes more significant in the control of simple motor tasks if the M1 is injured (Peters et al., 2018), indicating that the SMA is more involved in performing difficult tasks than M1. However, the “dis/connection” itself does not have any implication of the “control” capability, to precisely describe the motor control deficits and the ability of various brain regions in guiding the brain into easy or difficult states in response to the tasks. Therefore, in this study, we employed a novel “controllability” measure to specifically describe the “control” ability loss of the above motor regions in stroke patients.

Network control theory is an innovative and leading subfield of dynamic network theory that offers powerful engineering-based concepts to examine functional signaling in the networked systems (Gu et al., 2015). Traditional graph-based measurements such as node degree, betweenness centrality, and clustering coefficient, describe the local properties of the network architecture (Bullmore and Sporns, 2009). However, these locally static graph measures themselves do not have any implication to describe the “control” ability of the regions in controlling the brain state transition (Fang et al., 2021). Differently, controllability diagnostics are systematic-level measures that describe the capability of different brain regions in affecting the network dynamics and steering the brain into various easy or difficult to reach states (Betzel et al., 2016). For example, the modal controllability indicates the capability of a specific brain region in controlling the brain network system into difficult-to-reach states (Gu et al., 2015). In a control perspective, our results demonstrated that the modal controllability of SMA in stroke patients was significantly lower than healthy controls (Figure 4A), indicating the SMA showed less control ability to guide the brain network system into hard-to-reach states in stroke patients. Physiologically, as mentioned above, the SMA is more involved in performing cognitively demanding tasks and the disconnection of SMA is associated with motor control deficits of stroke patients (Peters et al., 2018). Instead of interpreting the lost capability of motor control performance based on the static graph measures (dis/connection), our results interpreted the specific “motor control” deficits of stroke patients with a particular systematic measure, “controllability”, to precisely describe the “motor control” ability loss in stroke patients. Specifically, our results indicated that the motor control deficits caused by stroke may due to the lost capability of SMA in steering the brain network system into cognitively demanding states. Prior study reported that a subject’s cognitive processing and set-shifting speed appears to be coded, to some degree, in the connectivity strength of bilateral intraparietal sulcus nodes of the ECN (Seeley et al., 2007). From a control point of view, our results showed that the capability of ECN to control the brain to enter some difficult states was lost in stroke patients (Figure 4B). This may explain the motor impairment of stroke patients in performing some control-demanding tasks that require higher level cognitive processing provided by ECN to complete the difficult tasks.

In this study, we also correlated the controllability values with the clinical scores (FM-UL). In our results, the baseline modal controllability of M1 showed significantly positive correlation with the baseline FM-UL scores (Figure 5A). Even though most studies hypothesized the M1 controlled movement at a simple level, some researches also demonstrated that the M1 may serve some complex function than originally hypothesized (Graziano, 2006). Our results further illustrated that the capability of M1 to steer the brain network system into some complex brain states that require a lot of cognitive effort may account for the motor reservation of stroke, and be utilized as biomarkers to predict the reservation of motor performance in stroke patients at baseline. Unfortunately, due to the limited sample size of patients who have both pre- and post-intervention EEG-fNIRS recordings, the changes of modal controllability could not significantly predict the changes of clinical scores, although high correlations were observed (Figure 5B). This will be improved as the immediate next step once we have more patients with the post-intervention.

In this study, we quantified the contribution of topological factor (node strength) to the variability in controllability in stroke patients and healthy subjects, respectively. As reported in previous study (Jeganathan et al., 2018), lower correlation between the node strength and the controllability measure indicated that other network features or factors may influence the nodes’ controllability. In our results, we showed that the correlation between node strength and controllability in stroke patients was lower than that of health subjects (Figure 3). This may indicate that the network alterations caused by stroke may break the underlying neural control patterns by increasing the effects of other network features in contributing to the normal control patterns.

While the current investigation provides a new perspective to interpret the specific motor control deficits in stroke patients, some limitations and drawbacks must be acknowledged. First and foremost, the sample size is relatively small in this study. Meanwhile, the clinical characteristics of patients are rather heterogeneous, such as lesion size, location, initial motor impairment (11–61), stroke phase (acute/subacute) and stroke subtype (cortical/subcortical). These variables could have certain effects on characterizing the behavioral and neurological outcomes. Besides, even though a high spatiotemporal resolution brain imaging approach was employed to reconstruct the source activities, the brain model utilized for each subject was from a common brain model, which may induce mild bias when estimating the cortical activities. The immediate next step will be collected the magnetic resonance imaging (MRI) data from those participants to construct the patient-specific brain model, to further increase the fNIRS-informed EEG source localization accuracy. Moreover, in this study, we only considered the EEG sources located in the cortical areas due to the shallow penetration depth of fNIRS (around 1–3 cm) in the cortex (Liu et al., 2015), but will be improved with the development of advanced neuroimaging techniques and algorithms. Additionally, the current study employed a simple linear network dynamic model, which remains to be improved to account for the nonlinear effect in future. Finally, as our experimental paradigm asked the subjects to perform motor control behaviors from a resting state, we assumed this is a difficult-to-reach process (compared to the brain states transition between resting to sleeping or resting to resting) that requires significant cognitive effort from the resting state (especially for stroke patients), which is consistent with the definition of modal controllability. In future studies, we may employ other brain controllability measurements such as average controllability and global controllability to investigate the control properties of the brain in stroke and other diseases.

## Conclusion

This study represents the first attempt to employ the network “controllability” diagnostic to specifically interpret the “motor control” deficits caused by stroke. In addition, the current study is also the first study to apply the brain controllability analysis based on the non-invasive, portal, and costless neuroimaging tools with a high spatiotemporal fNIRS-informed EEG source imaging strategy. The results demonstrated that the modal controllability of SMA and ECN were significantly decreased in stroke patients compared to healthy subjects, and the baseline modal controllability of M1 could be utilized to predict the clinical scores at baseline for stroke patients. The methodologies proposed in this study may be extended to investigate the cognitive/motor control deficits caused by other neurological or psychiatric diseases, and design neuromodulation strategies by employing the network control theory in an experimentally friendly manner.

## Data Availability

The dataset is available upon reasonable request to the corresponding author. Requests to access these datasets should be directed to yzhang94@uh.edu.
